# Mode of action of DNA-competitive small molecule inhibitors of tyrosyl DNA phosphodiesterase 2

**DOI:** 10.1042/BCJ20160180

**Published:** 2016-06-28

**Authors:** Peter Hornyak, Trevor Askwith, Sarah Walker, Emilia Komulainen, Michael Paradowski, Lewis E. Pennicott, Edward J. Bartlett, Nigel C. Brissett, Ali Raoof, Mandy Watson, Allan M. Jordan, Donald J. Ogilvie, Simon E. Ward, John R. Atack, Laurence H. Pearl, Keith W. Caldecott, Antony W. Oliver

**Affiliations:** * Genome Damage and Stability Centre, School of Life Sciences, University of Sussex, Falmer BN1 9RQ, U.K.; † Cancer Research UK DNA Repair Enzymes Group, Genome Damage and Stability Centre, School of Life Sciences, University of Sussex, Falmer BN1 9RQ, U.K.; ‡ Sussex Drug Discovery Centre, School of Life Sciences, University of Sussex, Falmer BN1 9QJ, U.K.; § Drug Discovery Unit, Cancer Research UK Manchester Institute, University of Manchester, Manchester M20 4BX, U.K.

**Keywords:** deoxyribonucleic acid (DNA) synthesis and repair, drug discovery and design, topoisomerases

## Abstract

Tyrosyl-DNA phosphodiesterase 2 (TDP2) is a 5′-tyrosyl DNA phosphodiesterase important for the repair of DNA adducts generated by non-productive (abortive) activity of topoisomerase II (TOP2). TDP2 facilitates therapeutic resistance to topoisomerase poisons, which are widely used in the treatment of a range of cancer types. Consequently, TDP2 is an interesting target for the development of small molecule inhibitors that could restore sensitivity to topoisomerase-directed therapies. Previous studies identified a class of deazaflavin-based molecules that showed inhibitory activity against TDP2 at therapeutically useful concentrations, but their mode of action was uncertain. We have confirmed that the deazaflavin series inhibits TDP2 enzyme activity in a fluorescence-based assay, suitable for high-throughput screen (HTS)-screening. We have gone on to determine crystal structures of these compounds bound to a ‘humanized’ form of murine TDP2. The structures reveal their novel mode of action as competitive ligands for the binding site of an incoming DNA substrate, and point the way to generating novel and potent inhibitors of TDP2.

## INTRODUCTION

Tyrosyl-DNA phosphodiesterase 2 (TDP2) {also known as TRAF and TNF receptor-associated gene (TTRAP) [1] or ETS1-associated protein II (EAPII) [2]} is a 5′-tyrosyl DNA phosphodiesterase that can remove the covalently-attached abortive complexes generated by DNA topoisomerase II (TOP2) when it is unable to complete its normal catalytic cycle [3,4]. TDP2 has also been co-opted by picornaviruses as the ‘unlinkase’ that releases nascent RNA from its VPg protein primer during viral replication [5].

TOP2 generates transient double-strand breaks (DSBs) in genomic DNA that relieve torsional stress introduced during transcription and replication [6]. The mechanism of DNA strand cleavage by TOP2 involves formation of a covalent reaction intermediate between the side chain of a catalytic tyrosine in the enzyme, and the 5′-phosphate at the site of the DNA-strand break. TOP2 functions as a dimer, and produces a staggered DSB, with the nicks in the two strands separated by 4 bp. Normally, following passage of a distal segment of duplex DNA through the break, TOP2 facilitates attack on the phosphotyrosyl bonds by the free 3′-OH at the nicks, resealing the break and releasing the enzyme. In some circumstances, TOP2 is unable to complete the reaction cycle, leaving it covalently attached to the DSB 5′-termini in a highly toxic, abortive, ‘cleavage complex’ that can severely disrupt replication and transcription. Fortunately, this type of DNA lesion is efficiently repaired, in part though the action of TDP2, which hydrolyses the phosphotyrosyl bond in a magnesium cofactor-dependent reaction, leaving ligatable 5′-phosphate moieties.

TOP2 ‘poisons’ such as doxorubicin (Adriamycin) are known to intercalate into DNA (PDB: 1D12; [7]); amsacrine (mAMSA) and etoposide (VP-1) have also been demonstrated to bind selectively at the nick sites of a TOP2–DNA complex (PDB: 4G0U, 3QX3; [8]) and prevent resealing of the strands. These interactions promote the cumulative formation of abortive TOP2–DNA complexes, leading ultimately to cell death in rapidly proliferating cells. Consequently, TOP2 poisons are potent anticancer agents [9], and are currently used in the clinic either as monotherapies, or as components of combination therapies for a range of tumour types [10–12]. However, the abortive complexes promoted by TOP2 poisons can be rescued by the action of TDP2, so that variations in cellular TDP2 activity (natural or tumour-specific) can influence the clinical efficacy of these drugs, and are therefore an important determinant of response to chemotherapy in the individual patient and tumour. Consequently, TDP2 has attracted interest as a target for small molecule inhibitors that might have utility in overcoming innate or acquired resistance to TOP2 poisons.

A series of potent TDP2 inhibitors based on a deazaflavin scaffold have previously been described, but their molecular mode of action could not be determined and the rational exploitation of structure–activity relationships (SARs) was consequently limited [13]. Using a ‘humanized’ mouse TDP2 expression construct, we have now determined X-ray crystal structures of the catalytic domain of TDP2 in complex with deazaflavin inhibitors, revealing the unusual molecular interactions underpinning their mode of action. These results provide a platform for structure-based drug design that will greatly assist the future development of small molecule inhibitors of human TDP2.

## MATERIALS AND METHODS

### Expression constructs

#### hTDP2^CAT^

DNA encoding the catalytic domain of human TDP2 (amino acids 113–362) was cloned into pET-28a (Novagen, Merck Chemicals). Using standard site-directed mutagenesis techniques, a single-point mutant–C273S–was subsequently introduced into this expression construct; improving the behaviour of the recombinant protein, qualitatively with respect to both solubility and aggregation.

#### mTDP2^CAT^/m2hTDP2^CAT^

Synthetic genes codon-optimized for *Escherichia coli* expression were sub-cloned into an in-house modified form of pET-15b, which encodes an N-terminal His-small ubiquitin-like modifier (SUMO) affinity/solubility tag that is cleavable by sentrin-specific protease 1 (SENP1) protease (Genscript). The mTDP2^CAT^ expression construct encodes amino acids Leu^118^–Leu^370^ of the full-length gene. The m2hTDP2^CAT^ construct encodes the same amino acid range, but with the following series of mutations: E242G, Q278R, Y321C and H323L.

### Protein expression and purification

#### hTDP2^CAT^

Transformed colonies of *E. coli* strain B834 (DE3) were used to inoculate a 250 ml flask containing 50 ml of Turbo broth (Molecular Dimensions) supplemented with kanamycin (50 μg/ml). The inoculated culture was grown at 37°C, at 220 rpm, in an orbital shaking incubator until the absorbance at 600 nm reached approximately 1.5. The culture was then stored overnight at 4°C.

The following day, 20 ml of the overnight culture was used to inoculate a 2 litre flask containing 1 litre of Turbo broth supplemented with antibiotic as before. Cultures were grown at 37°C, at 220 rpm, in an orbital shaking incubator until the absorbance at 600 nm again reached approximately 1.5. Recombinant protein expression was then induced by the addition of 0.4 mM IPTG, and the culture incubated for a further 3.5 h at a reduced temperature of 30°C. Cells were then harvested by centrifugation at 7548 ***g***, for 10 minutes at 4°C, and the resulting cell pellet stored at −80°C until required.

The cell pellet arising from 4 litres of culture was resuspended in buffer A: 50 mM HEPES/NaOH, pH 7.5, 250 mM NaCl, 10 mM imidazole, 0.5 mM TCEP, supplemented with protease inhibitors (Roche), then disrupted by sonication, and the resulting lysate clarified by high-speed centrifugation at 40000 ***g***, for 60 min at 4°C.

The supernatant was applied to a batch/gravity flow column containing 10 ml of Talon resin (TaKaRa Bio) pre-equilibrated in buffer A. After incubation (with rolling) for a period of 1 h at 4°C, any unbound material was removed by sequential washes with buffer A. Retained protein was then eluted by application of the same buffer supplemented with 300 mM imidazole, pH 7.5.

The eluate was diluted, in order to reduce the salt concentration to <150 mM NaCl, and then applied to a 5 ml HiTrap Heparin HP column (GE Healthcare) equilibrated in buffer B: 50 mM HEPES/NaOH, pH 7.5, 150 mM NaCl, 1 mM TCEP. Unbound material was first removed by application of five column volumes of the same buffer. Any retained protein was eluted, through application of a linear salt gradient (0.15–1 M NaCl) to the column.

A HiLoad 16/600 Superdex 75 pg size exclusion column (GE Healthcare) was used to purify hTDP2-CAT to homogeneity in 20 mM HEPES/NaOH, pH 7.5, 300 mM NaCl, 0.5 mM TCEP.

#### mTDP2^CAT^/m2hTDP2^CAT^

A similar protocol was also used to purify both mTDP2^CAT^ and m2hTDP2^CAT^, with the following alterations: *E. coli* strain: Rosetta 2(DE3)pLysS (Novagen); antibiotic selection: 50 μg/ml ampicillin/34 μg/ml chloramphenicol. The His-SUMO affinity/solubility tag was cleaved overnight at 4°C by the addition of SENP1 protease, after the initial Talon capture step.

### Thermal denaturation

For thermal denaturation, samples containing protein at 1.7 μM and 5 x SYPRO Orange (diluted from a 5000 x stock supplied in DMSO; Sigma–Aldrich) were prepared in sample buffer: 50 mM HEPES/NaOH, pH 7.5, 300 mM NaCl, 0.5 mM TCEP. Either DMSO alone or compound [dissolved in 100% (v/v) DMSO at its maximum solubility] was then added; producing a final concentration of 3% (v/v) DMSO. Denaturation curves were monitored in 96-well PCR plates using a Roche LightCycler 480 II, with 465 nm and 580 nm filters for excitation and emission wavelengths respectively. The programme was as follows: 1 min at 20°C, followed by a continuous increment of 0.03°C/s to a final temperature of 85°C. Temperature midpoints (*T*_m_) for each folded to unfolded transition were determined by non-linear regression fitting of a modified Boltzmann model to normalized data in Prism 6.0 (version 6.0h, GraphPad Software).

$$Y=\left(a_{n}X+b_{n}\right)+\frac{\left(a_{d}X+b_{d}\right)-\left(a_{n}X+b_{n}\right)}{1+e^{\frac{T_{m}-X}{m}}}$$

where *a*_n_ and *a*_d_ are the slopes and *b*_n_ and *b*_d_ the *y*-intercepts, of the native and denatured baselines respectively. *T*_m_ is the temperature midpoint of the transition from native to denatured states and *m* represents a generic slope factor.

### Fluorescence-based enzyme activity assay

Substrate oligonucleotide containing a 5′-phosphotyrosine (5′-Y-GATCTAAAAGACT-3′) conjugated to FITC was purchased from Midland Certified Reagents.

This assay is a modified version of that reported by [14]. Briefly, enzyme assays were run in black 384-well plates, in 50 mM Tris/HCl, pH 8.0, 10 mM MgCl_2_, 80 mM KCl, 0.05% (v/v) Tween-20, 1 mM DTT. Fifty picomoles of hTDP2^CAT^ were combined with 25 nM substrate oligonucleotide, in a final volume of 15 μl, for a period of 10 min, before addition of quench reagents (Gyrasol Technologies; sensor diluted 1:15 with enhancer buffer). Fluorescence was subsequently measured in a PHERAstar multimode plate reader (BMG Labtech GmbH) with an excitation wavelength of 490 nm, and an emission wavelength of 520 nm. The built-in analysis tools of Prism 6.0 was used to examine all experimental data.

### Gel-based enzyme activity assay

This assay is a modified version of that reported by [3,4]. Briefly, recombinant TDP2^CAT^ proteins were diluted with reaction buffer: 50 mM Tris/HCl, pH 7.5, 50 mM KCl, 1 mM MgCl_2_, 1 mM DTT to produce final concentrations of 1 nM (for hTDP2^CAT^ and m2hTDP2^CAT^) or 3 nM (for mTDP2^CAT^) in a volume of 16.2 μl–either in the presence or absence of compound [50 nM for hTDP2^CAT^ and m2hTDP2^CAT^, 150 nM for mTDP2^CAT^; 1% (v/v) final DMSO concentration]. To start the reaction, 1.8 μl of radiolabelled 5′-phosphotyrosyl DNA substrate was added to the mixture, then incubated at 37°C. At 2, 5 and 10 min time-points, 6 μl of the reaction mixture was withdrawn, and stopped by the addition of formamide loading buffer. Samples were then analysed by denaturing PAGE, visualized by phosphorimager, with intensities of substrate and product bands measured by GelAnalyzer2010 software (http://www.gelanalyzer.com).

### Crystallization and data collection

#### hTDP2^CAT^

Crystals of hTDP2^CAT^ were grown at 20°C in 24-well hanging-drop vapour-diffusion plates, mixing 1 μl of protein at 7.5 mg/ml with 1 μl of 100 mM Bis–Tris propane, pH 7.0, 0.5 M NaCl, 0.05 M magnesium acetate, 1.5% (v/v) trimethylamine N-oxide, equilibrated against 500 μl of the same solution. Crystals were swiped successively through buffers containing increasing concentrations of cryo-protectant, before being plunged into liquid nitrogen; 30% (v/v) glycerol was sufficient to prevent ice formation.

Diffraction data to 3.1 Å (1 Å=0.1 nm) resolution were collected at the Diamond Light Source (DLS) on beamline I04. Crystals grew in space group P3_1_ 2 1, with two molecules of hTDP2^CAT^ comprising the asymmetric unit.

#### hTDP2^CAT^/163

Crystals of hTDP2^CAT^/163 were grown at 20°C in 24-well hanging-drop vapour-diffusion plates, mixing 1 μl of complex (1:2.7 molar ratio protein: compound) at approximately 7.5 mg/ml, with 1 μl of 1.2 M D/L-malic acid pH 7.0, 0.1 M Bis–Tris propane pH 7.0, 3% (v/v) DMSO, equilibrated against 500 μl of the same solution. Crystals were swiped successively through buffers containing increasing concentrations of cryo-protectant, before being plunged into liquid nitrogen; 40% (w/v) sucrose was sufficient to prevent ice formation.

Diffraction data to 3.4 Å resolution were collected at the Diamond Light Source (DLS) on beamline I04. Crystals grew in space group P6_5_ 2 2, with a single molecule of hTDP2^CAT^ in complex with 163 comprising the asymmetric unit.

#### m2hTDP2^CAT^/163

Crystals of m2hTDP2^CAT^/163 were grown at 20°C in 24-well hanging-drop vapour-diffusion plates, mixing 1 μl of complex (1:2.7 molar ratio protein: compound) at approximately 7.5 mg/ml, with 1 μl of 0.1 M Bis–Tris propane pH 7.5, 0.2 M sodium citrate, 20% (w/v) PEG3350, 0.3% (v/v) DMSO, equilibrated against 500 μl of the same solution. Crystals were swiped successively through buffers containing increasing concentrations of cryo-protectant, before being plunged into liquid nitrogen; 8% (v/v) glycerol+5% (w/v) glucose was sufficient to prevent ice formation.

Diffraction data to 1.8 Å resolution were collected at the Diamond Light Source (DLS) on beamline I04. Crystals grew in space group *P*2_1_, with two molecules of m2hTDP2^CAT^, both in complex with 163, comprising the asymmetric unit.

#### m2hTDP2^CAT^/148

Crystals of m2hTDP2^CAT^/148 were grown at 20°C in 24-well hanging-drop vapour-diffusion plates, mixing 1 μl of complex (1:2.7 molar ratio protein: compound) at approximately 7.5 mg/ml, with 1 μl of 0.1 M Bis–Tris propane pH 6.5, 0.2 M sodium tartrate, 20% (w/v) PEG3350, 0.3% (v/v) DMSO, equilibrated against 500 μl of the same solution. Crystals were swiped successively through buffers containing increasing concentrations of cryo-protectant, before being plunged into liquid nitrogen; 8% (v/v) glycerol+5% (w/v) glucose was sufficient to prevent ice formation.

Diffraction data to 1.7 Å resolution were collected at the Diamond Light Source (DLS) on beamline I04. Crystals grew in space group *P*2_1_, with two molecules of m2hTDP2^CAT^, both in complex with 148, comprising the asymmetric unit.

### Phasing, model building and refinement

All diffraction data were collected at 100 K. Data were integrated using the software package XDS [15], and then processed using the Pointless/Aimless/Ctruncate pipeline of the CCP4 software suite [16–19]. Phases were obtained by molecular replacement, using PHASER with 4GYZ (mTDP2-CAT) as a search model [20]. An iterative combination of manual building in Coot [21] and refinement with either phenix.refine [22] or BUSTER [23] produced the final models.

### Compounds

Compounds either were provided by the Cancer Research UK Manchester Institute Drug Discovery Unit or were re-synthesized in-house by LP or M.P., following the published protocols [13].

## RESULTS

### Structure of the human TDP2 catalytic domain

**Figure 1:**
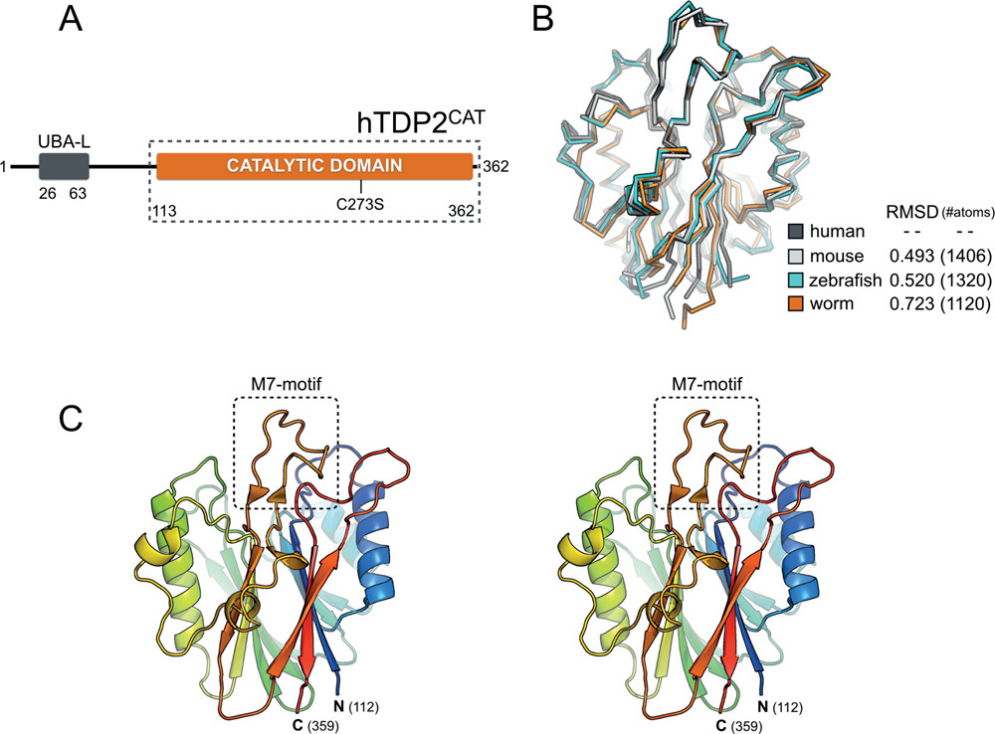
Structure of the catalytic domain of human TDP2 (**A**) Schematic representation of human TDP2, highlighting the relative positions of its component domains; where UBA-L is Ubiquitin associated-like domain. The amino acid boundaries of the hTDP2^CAT^ expression construct are indicated by the dotted box. The position of the C273S mutation introduced to improve protein solubility is also indicated. (**B**) Superposition of TDP2 catalytic domains. Structures for human, mouse, worm and zebrafish TDP2^CAT^ are each shown in ribbon representation. RMSD values, over the indicated number of equivalent atom positions, were calculated using PyMOL (www.pymol.org). (**C**) Stereo-view of the X-ray crystal structure of hTDP2^CAT^. Molecular cartoons are coloured from blue➔red, from the visible N- to C-terminus. The position of the M7-loop or motif is additionally highlighted.

We obtained crystals of the catalytic domain of human TDP2 (Figure 1A; hTDP2^CAT^–residues 113–362), in its unliganded form, using protein expressed in *E. coli* and purified using standard chromatographic procedures (see ‘Materials and Methods’). The expression construct contains a single-point mutant–C273S–which removes a potentially reactive surface cysteine, and that in our hands significantly improved the expression levels and solubility of the recombinant protein.

The structure of hTDP2^CAT^ was determined by molecular replacement, with the catalytic domain of mouse TDP2 as a search model (PDB: 4GYZ). However, crystals of hTDP2^CAT^ typically diffracted both poorly and anisotropically. Through iterative screening of many crystals, we were eventually able to collect a dataset that could be truncated isotropically to a maximum resolution of 3.1 Å (see Table 1). The fold of the human protein is highly similar to that of mouse [24], worm [25], and zebrafish [25] (Figures 1B and 1C) but with some important differences–see expanded description below.

**Table 1:** Data collection and refinement statistics *Values in parentheses are for highest-resolution shell. ^‡^Data isotropic to this resolution. ^§^PDB entry used as ‘reference model’ during refinement.

	hTDP2-CAT	hTDP2-CAT+**163**	m2hTDP2-CAT+**163**	m2hTDP2-CAT+**148**
	PDB: 5J3P	PDB: 5J3S	PDB: 5J3Z	PDB: 5J42
Data collection				
Space group	*P*3_1_21	*P*6_5_22	*P*2_1_	*P*2_1_
Cell dimensions				
*a*,*b*,*c* (Å)	92.54, 92.54, 119.07	68.52, 68.52, 209.51	61.00, 42.80, 108.75	61.05, 42.87, 109.05
*α*, *β*, *γ* (°)	90, 90,120	90, 90, 120	90, 94.15, 90	90, 94.05, 90
Resolution (Å)	47.79–3.10 (3.31–3.10)*	45.22–3.40 (3.67–3.40)	42.8–1.80 (1.84–1.80)	42.07–1.70 (1.73-1.70)
*R_merge_*	0.133 (0.731)	0.256 (1.876)	0.051 (0.166)	0.043 (0.810)
*R_meas_*	0.170 (0.932)	0.299 (2.185)	0.072 (0.233)	0.061 (1.133)
*R*_pim_	0.103 (0.571)	0.168 (1.111)	0.050 (0.164)	0.042 (0.791)
Mn*I*/ σ*I*	9.6 (1.7)	6.4 (1.1)	9.7 (2.0)	16.3 (1.0)
CC^1^/_2_	1.00 (0.94)	0.99 (0.54)	0.99 (0.87)	1.00 (0.60)
Completeness (%)	99.8 (99.7)	99.9 (100)	98.0 (79.7)	99.2 (98.9)
Multiplicity	4.8 (4.9)	6.3 (6.8)	2.9 (2.1)	3.1 (3.2)
Refinement				
Resolution (Å)	47.79–3.10^‡^	45.22–3.40^‡^	42.8–1.80	42.07–1.70
No. unique reflections	11128	7545	51379	112934
*R*_work_/*R*_free_	0.26/0.30	0.25/0.27	0.17/0.20	0.16/0.20
No. of atoms				
Protein	3553	1732	3992	3968
Ligand/ion	8	29	168	142
Water	7	–	604	693
*B*-factors				
Protein	98.2	119.9	23.5	26.6
Ligand/ion	84.3	78.7	42.1	30.9
Water	43.4	–	40.7	44.9
Root-mean-square (R.m.s.) deviations				
Bond lengths (Å)	0.002	0.001	0.008	0.020
Bond angles (°)	0.551	0.435	0.98	1.654
Reference model^§^	4GYZ	4GYZ	N/A	N/A
Ramachandran				
Outliers (%)	5	2	0	0
Favoured (%)	92.4	92.6	97.9	97.9
MolProbity score	1.8	1.9	1.28	1.50

### Deazaflavin inhibitors of TDP2

**Figure 2:**
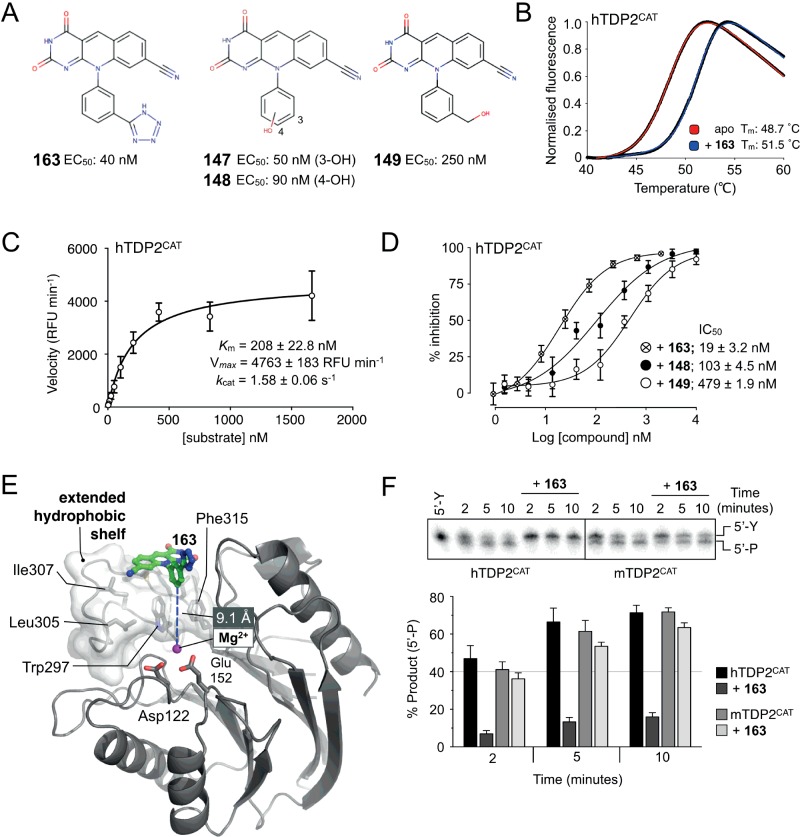
Small molecule Inhibitors of human TDP2 (**A**) Chemical drawings of four deazaflavin-based inhibitors of human TDP2, as described by Raoof et al. [13]. Compound numbering and EC_50_ values are as previously reported. (**B**) Thermal denaturation profiles for hTDP2^CAT^ in the absence or presence of 300 μM 163. The experimental data (data points coloured in black) were fitted with a modified Boltzmann model to obtain the indicated *T*_m_ for the apo- (red curve) or liganded (blue curve) forms of the protein. (**C**) Michaelis–Menten curve for hTDP2^CAT^ using the fluorescence-based enzyme assay. Fifty picomoles of hTDP2^CAT^ were incubated with substrate oligonucleotide for a period of 10 min, before the addition of Gyrasol reagents. Experimental data were fitted with a standard Michaelis–Menten equation (Prism 6, Graphpad software) in order to determine *K*_m_ and *V*_max_ parameters. Data points are the mean of three independent experiments, with error bars representing one S.D. (**D**) Dose–response curves for inhibition of hTDP2^CAT^ by increasing concentrations of the indicated deazaflavin. See associated key for details, and IC_50_ values determined. Data points are the mean of six replicates, with error bars representing one S.D. (**E**) Molecular cartoon highlighting the binding position of 163, relative to the active site of hTDP2^CAT^. The compound packs against an extended hydrophobic shelf (light grey molecular surface+stick representation) formed by the side chains of Trp^297^, Thr^299^, Leu^305^, Ile^307^, Cys^311^, Leu^313^ and Phe^315^. The compound binds at a distance (>9 Å) from the active site of the enzyme, as indicated by the side chains of Asp^122^ and Glu^152^, and the magnesium cofactor (purple sphere). (**F**) Top: representative image for the gel-based enzyme activity assay. hTDP2^CAT^ efficiently catalyses the conversion of a 5′-phosphotyrosylated (5′-Y) DNA duplex substrate to a 5′-phosphorylated product (5′-P) with time. Although 163 clearly inhibits the human enzyme, it is ineffective against the mouse equivalent. Bottom: quantification of gel-based assay. Data represent the mean of three independent experiments, with errors bars representing one S.D.

A series of small molecule deazaflavin inhibitors of human TDP2 have previously been described [13], with examples showing *in vitro* biochemical activity against the isolated enzyme in the nanomolar concentration range (Figure 2A). However, the precise mode of action of these compounds was unclear, and in the absence of an experimentally determined binding site, the SARs were difficult to rationalize.

As the original high-throughput screen (HTS) screens were performed with full-length human TDP2, we first sought to confirm binding of these compounds to hTDP2^CAT^ by thermal denaturation [26]. Using the most potent reported deazaflavin–compound 163 [13], we observed a shift in the temperature midpoint (*T*_m_) of hTDP2^CAT^ from 48.7°C in the unliganded state, to 51.5°C in the presence of compound 163 at a concentration of 300 μM. These results are indicative of moderately tight binding of 163 to hTDP2^CAT^ (Figure 2B).

We also confirmed inhibition of catalytic activity by three deazaflavin compounds, by using a modified form of a fluorescence-based assay [14]. A phosphotyrosyl moiety conjugated to FITC was readily removed from the 5′ end of a single-stranded oligonucleotide substrate by hTDP2^CAT^ in a dose-dependent manner (Supplementary Figures S1A and S1B). We could subsequently determine *K*_m_ for the oligonucleotide substrate (208±23 nM), *K*_cat_ for the reaction under the experimental conditions tested (1.58±0.06 s^−1^; Figure 2C), as well as IC_50_ values for each of the deazaflavins; 163: 19, 148: 103, 149: 489 nM (Figure 2D). The IC_50_ rank order is in agreement with the previously reported EC_50_ values, which used full-length human TDP2 and BIOMOL Green as a detection/quantification agent [13].

### Low-resolution co-crystal structure of hTDP2^CAT^ bound to 163

As with the apo-hTDP2^CAT^ crystals, co-crystals with 163 presented severe problems with anisotropy during data collection. Again, multiple crystals were screened before a dataset that could be truncated isotropically, to a maximum resolution of 3.4 Å, was obtained (Table 1). Despite the low-resolution, which prevents detailed characterization of specific protein-drug interactions, clear difference electron density maps identified the binding site and general pose of this class of inhibitor bound to TDP2 (Supplementary Figure S2).

The experimentally determined binding-mode of 163 is surprisingly different from that predicted by molecular docking studies [13], in which the pyrimido-dione ring of the tricyclic core, was hypothesized to bind to the magnesium cofactor–required for catalytic activity of TDP2–and made few (if any) hydrogen bonds directly to the protein.

Instead, our data indicate that the tricyclic deazaflavin core of 163 unexpectedly packs against an extended hydrophobic patch, formed by the side chains of Trp^297^, Thr^299^, Leu^305^, Ile^307^, Cys^311^, Leu^313^ and Phe^315^, which lies at the mouth of the channel leading to the active site, more than 9 Å from the catalytic Mg^2+^ ion bound by the carboxyl side chains of Asp^122^ and Glu^152^ (Figure 2E).

Due to the persistently poor crystallization behaviour of the human protein, and the need to characterize fully this unusual and unexpected binding mode of the deazaflavin inhibitor, we switched species and used the murine form of the enzyme (mTDP2^CAT^), which yielded well-behaved crystals diffracting to high-resolution; as in previous studies [24]. As a preliminary experiment, we tested the ability of 163 to inhibit mTDP2^CAT^ in a gel-based assay. Unexpectedly, although 163 readily inhibited the catalytic activity of hTDP2^CAT^–preventing conversion of 5′-phosphotyrosyl termini to 5′-phosphates on a DNA duplex substrate–it failed to inhibit the mouse protein (Figure 2F).

**Figure 3:**
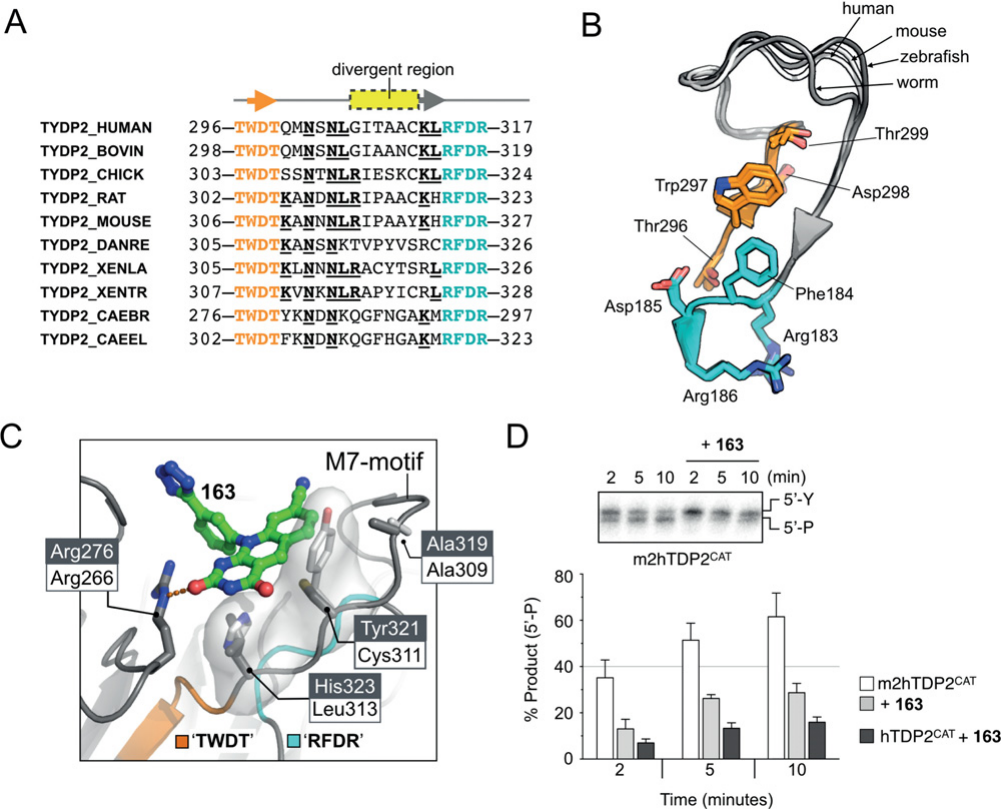
The M7-motif of TDP2 (**A**) Multiple amino acid sequence alignment for the M7-motif of TDP2. This region is flanked by the two highly conserved ‘TWDT’ (coloured orange) and ‘RFDR’ (coloured cyan) motifs. Amino acid boundaries and UniProt accession codes are as indicated. Partially conserved amino acids are additionally identified by the bold, underlined text. (**B**) Superposition of the M7-motif taken from X-ray crystal structures of human (the present study), mouse (PDB:4GYZ), zebrafish (4F1H) and worm TDP2 (4FVA). The spatial arrangement of the ‘TWDT’ and ‘RFDR’ motifs is highly conserved. The loop connecting these two elements is conserved in length, but varies somewhat in conformation. For clarity, numbering is only shown for the human protein. (**C**) Molecular cartoon of 163 in complex with hTDP2^CAT^ highlighting the position of two amino acid residues (Leu^313^ and Cys^311^; black text on white background) that are not conserved in the M7-motif of mouse TDP2 (equivalent residues His^323^ and Tyr^231^; white text on grey background), but are essential for compound binding. (**D**) Top: representative image for the gel-based enzyme activity assay using m2hTDP2^CAT^. The introduction of four amino acid changes into mTDP2^CAT^–to generate m2hTDP2^CAT^–restores the inhibitory activity of 163. Labelling as per Figure 2(D). Bottom: quantification of gel-based assay. Data represent the mean of three independent experiments, with errors bars representing one S.D.

Comparison of the mouse and human TDP2 amino acid sequences revealed substantial differences in a segment of polypeptide, denoted the ‘M7-motif’ [24], delimited by highly conserved ‘TWDT’ (amino acids 296–299 in humans) and ‘RFDR’ (amino acids 314–317) motifs. A multiple amino acid sequence alignment of this region (Figure 3A) shows it to be conserved in length (14 residues) among different organisms, but poorly conserved in amino acid sequence and somewhat variable in conformation (Figure 3B). Significantly, the M7-motif maps to the binding site we observe for 163 in hTDP2^CAT^, where human TDP2 residues Cys^311^ and Leu^313^, along with Ala^309^, coalesce to form a ‘hydrophobic-shelf’ on which the deazaflavin core of 163 sits. Replacement of Cys^311^ and Leu^313^ in hTDP2 with the equivalent residues Tyr^321^ and His^323^ in mTDP2 disrupts this hydrophobic-shelf and explains the resistance of the mouse enzyme to the deazaflavin-based inhibitors (Figure 3C).

### Development of a protein surrogate: m2hTDP2^CAT^

Although hTDP2^CAT^ is effectively inhibited by 163 and related deazaflavins, its poor crystallographic behaviour greatly limits the degree to which structure-led approaches can be used in order to optimize these compounds further for clinical development. Conversely, mTDP2^CAT^, although crystallographically well-behaved and able to yield high-resolution structures, does not bind deazaflavin inhibitors due to sequence differences in the M7-motif.

To overcome these problems, we designed a series of mutations in the M7-motif and associated structure, designed to ‘humanize’ the inhibitor-binding site of mTDP2 while retaining the favourable murine surface that facilitates well-ordered crystals. Rather than exchange the entire mouse M7-motif for that of the human protein, we decided as an initial approach only to ‘humanize’ the amino acids expected to be in the proximity of the bound compound, and not those which would simply point towards solvent. We therefore created the expression construct m2hTDP2^CAT^ (m2h=mouse-‘to’-human) by mutating the following mouse residues to their human equivalent: E242G, Q278R, Y321C and H323L.

The m2hTDP2^CAT^ protein retained full phosphodiesterase activity, and consistent with our prediction that the resistance of the mouse protein to deazaflavins was due to the variant M7-motif, this activity in m2hTDP2^CAT^ was inhibited by 163 (Figure 3D). As no further optimization of the ‘humanized’ construct was therefore required, m2hTDP2^CAT^ was used for all subsequent structural analyses.

### High-resolution co-crystal structure of m2hTDP2^CAT^ bound to 163

**Figure 4:**
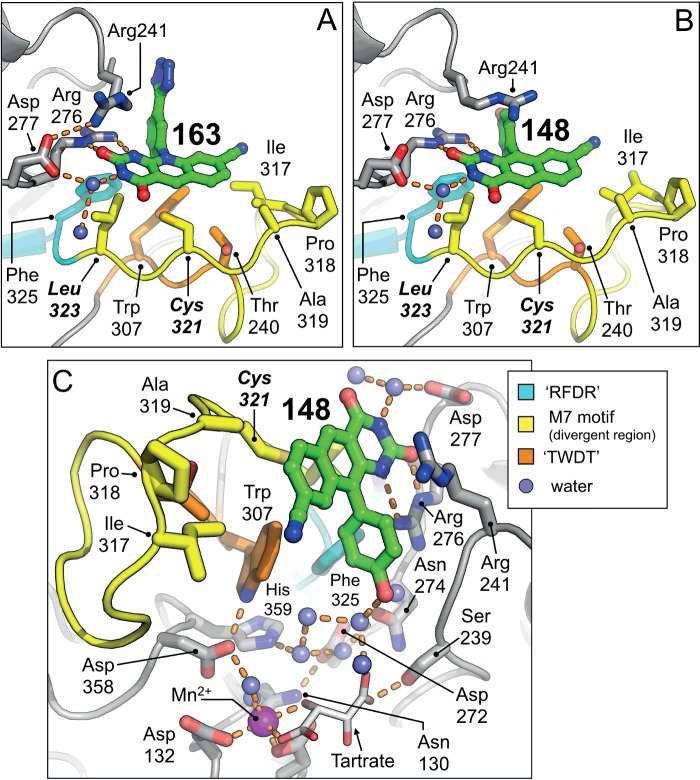
Molecular interactions of 163 and 148 with m2hTDP2^CAT^ (**A**) Molecular interactions made by 163. (**B**) Molecular interactions made by 148. (**C**) The *p*-hydroxyl of the 148 pendant group makes a series of water-mediated interactions with the side chain of Asp^272^ and Asn^274^, in close proximity to the active site of the enzyme. Molecular cartoons are shown throughout, with selected side chains and compounds shown in stick representation. Amino acids labelled in bold italic text, represent those that have been mutated from their mouse counterpart, in order to generate m2hTDP2^CAT^. Please see associated key for details of the colouring scheme.

We were able to validate our approach, by determining the X-ray crystal structure of m2hTDP^CAT^ bound to 163, at a resolution of 1.8 Å (Table 1). Superposition of the low-resolution hTDP2^CAT^ and high-resolution m2hTDP2^CAT^ structures in complex with 163**,** confirms that the binding site is conserved across both proteins (Supplementary Figure S3). However, the higher resolution data obtained with the m2hTDP2^CAT^ surrogate, allows a far more detailed characterization of the interaction of the inhibitor with the TDP2 catalytic domain (Figure 4A and Supplementary Figure S2).

The guanidinium head group of Arg^276^ (all numbering is for the mouse protein, the equivalent in human is calculated by subtraction of 10) is involved in an extensive hydrogen-bond network, including both carbonyl and nitrogen groups of the 163 pyrimido-dione ring, as well as the backbone carbonyl and side chain (via a water molecule) of Asp^277^. As in the fully human protein, amino acids of the M7-motif coalesce to form the ‘hydrophobic-shelf’, which permits extensive van der Waals interactions with the tricyclic deazaflavin core; these residues include Trp^307^, Ile^317^, Ala^319^, *Cys^321^* (text in italics represent a ‘humanized’ amino acid residue), *Leu^323^* and Phe^325^ (Figure 3A). The tetrazole R_1_ substituent of 163, is directed towards solvent, but packs against the carbon-atoms of the Arg^241^ side chain, providing additional van der Waals interactions.

### High-resolution co-crystal structure of m2hTDP2^CAT^ bound to 148

Raoof et al. [13] also described and characterized two hydroxyphenyl substituents at the R_1_ position of the deazaflavin core, in combination with the cyano group at the R_3_ position: compound 147 (3-hydroxyphenol) and 148 (4-hydroxyphenol) with reported EC_50_ values of 50 nM and 90 nM respectively (Figure 2A). We were successful in obtaining diffracting crystals of 148 in complex with m2hTDP2^CAT^, determining the structure at a resolution of 1.7 Å (Table 1). In this instance, we also supplemented the purification buffers with manganese chloride (iso-structural with magnesium, but readily discernible in electron density maps) in order to more fully define any interaction of the inhibitors with the metal-dependent catalytic centre of the enzyme.

In the 148 complex, the side chains of Arg^276^ and Asp^277^ make the same set of interactions with the carbonyl and nitrogen groups of the pyrimido-dione ring, as with 163. However, the side chain of Arg^241^ occupies a different position, breaking its hydrogen bond interaction with Asp^277^, and instead packing in a π–π interaction against the face of the tricyclic core of the deazaflavin (Figure 4B and Supplementary Figure S2). Interestingly, the *p*-hydroxyl group of the R_1_ substituent in 148, points directly towards the catalytic core of the protein and makes a series of water-mediated contacts to the side chains of Asp^272^ and Asn^274^. Electron density maps also clearly showed the position of a tartrate molecule (picked up from the crystallization mother liquor), which is hydrogen-bonded to the side chain of Ser^239^, as well as an extensive series of water-mediated interactions with several amino acid residues surrounding the catalytic centre. The tartrate molecule also completes the octahedral coordination of the Mn^2+^ ion (marking the position of the catalytic Mg^2+^ cofactor) (Figure 4C). Moreover, its position marks a potentially useful ‘molecular interaction path’ that could be utilized in future iterations of TDP2 inhibitor compounds.

### Mode of action of deazaflavin inhibitors of TDP2

Several structures for the catalytic domain of TDP2 in complex with nucleic acid have been reported [24,25]. However, there is disagreement between these structures regarding the path that the bound DNA follows across the protein surface; potentially as a result of the different substrates used (double compared with single-stranded) or possibly due to different crystal packing interactions.

**Figure 5:**
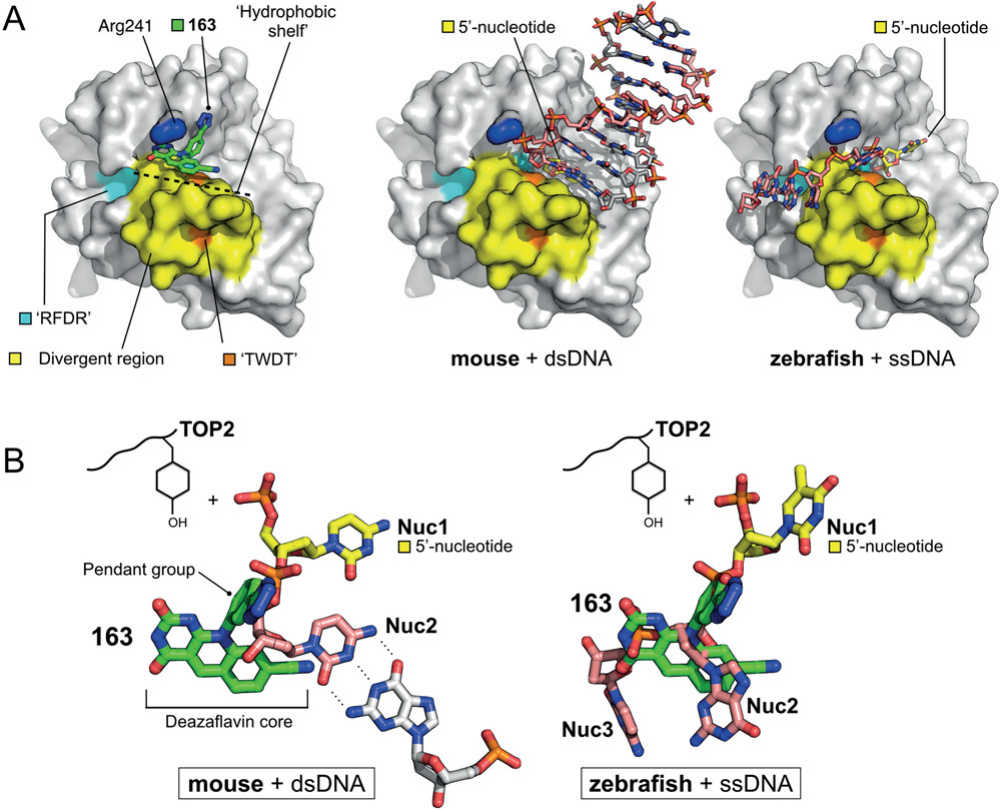
Deazaflavin binding relative to bound DNA (**A**) The deazaflavin-scaffold interacts with the ‘hydrophobic shelf’ formed by residues of the M7-motif. Molecular surface representations for: (left) m2hTDP2^CAT^ in complex with 163; (middle) mouse TDP2^CAT^ in complex with a double-stranded product DNA (PDB: 4GZ1); (right) zebrafish TDP2^CAT^ in complex with a single-stranded product DNA (PDB: 4F1H). (**B**) Superposition of structures reveals that the first two nucleotides of either product DNA (Nuc1, Nuc2) occupy highly similar positions when bound to TDP2. The dezaflavins occupy a position equivalent to Nuc2, with the pendant group sitting at the position of the 5′-phosphate, and the deazaflavin core at those of the ribose and base moieties. In both panels, the carbons of the previously modified 5′ nucleotide are coloured yellow, and those of the deazaflavin 163 in green.

However, taking PDB entries 4GZ1 and 4F1H as representative examples of the two different modes of TDP2–product DNA interaction, we note that although the DNA trajectories are different (Figure 5A), the position of the first (Nuc1) and second nucleotides (Nuc2) of the previously modified strand are highly similar in both structures (Figure 5B).

Regardless of which of these TDP2–product DNA structures actually represents a physiologically relevant binding mode, comparison with the structures reported here shows that the deazaflavins occupy a position equivalent to that of Nuc2, with the pendant group sitting at the position of the 5′-phosphate, and the deazaflavin core at the position of the ribose and base moieties.

## DISCUSSION

The proteins that constitute the DNA damage response (DDR) are increasingly being recognized as attractive targets for the development of new drugs, which exploit the inherent genomic instability found in many cancers [27,28]. This field has been galvanized by the approval of olaparib as a monotherapy for the treatment of specific subsets of patients with advanced ovarian cancer. Olaparib is an inhibitor of the DNA damage sensor poly(ADP-ribose) polymerase 1 (PARP1), which exploits cancer-associated genetic defects in one DNA repair system (homologous recombination-mediated DSB repair) by inhibiting a second system (single-strand break repair) to achieve potent and selective ‘synthetic lethality’ against tumour cells [29].

DDR enzymes that utilize cofactors–such as PARPs or protein kinases for example–fall within the traditional canon of drug development targets; i.e. where a specific cofactor-competitive inhibitor can be readily found. However, many biologically interesting DNA repair ‘targets’ for cancer drug discovery lack such cofactors and instead act directly on DNA. These present the challenge of discovering compounds capable of out-competing an often substantial protein–DNA interaction potentially dominated by highly polar interactions with the negatively-charged sugar-phosphate backbone.

The discovery of deazflavins as TDP2 inhibitors [13] highlighted the possibility that uncharged and ‘drug-like’ competitive inhibitors of DNA binding to a DNA repair enzyme, could be found and developed. However, the mode of action of these was unclear, and limited further optimization of these compounds towards clinical development. The structural analysis we describe here reveals a very unusual binding mode in which the deazaflavin core and pendant groups act as nucleoside mimetics, binding to the hydrophobic ‘shelf’ that can support the exposed face of the last base-paired nucleotide at the transition from double-stranded to single-stranded DNA in the mouse TDP2^CAT^–product DNA structure (PDB: 4GZ1). Although this deazaflavin series is specific for TDP2, many enzymes involved in DNA repair and manipulation engage with a dsDNA–ssDNA transition, and structural studies of their complexes with DNA reveal the presence of functionally equivalent ‘shelf’ structures that could also provide binding sites for DNA-competitive small ligands [30–32] that could have utility as selective inhibitors of the DNA repair pathways such enzymes mediate.

Interestingly, TDP2 has previously also been identified as a host factor required for the replication of picornaviruses in mammalian cells [5]; encoding the essential ‘unlinkase’, required to cleave a unique covalent interaction made between the virally-encoded protein VPg and RNAs produced by the virion. It is therefore feasible that small molecule inhibitors of TDP2 may also find utility as novel antiviral agents, for the treatment of picornoviral infections; which includes rhinovirus and enterovirus pathogens, both of which produce high morbidity rates in infants and children [5,33].

It is worth noting here (as previously reported [13]) that deazaflavin-based compounds are, in themselves, highly unlikely to be suitable as anticancer agents; in most part due to the general inability of the chemical scaffold to penetrate the cell membrane of mammalian cells. However, with the data gleaned from the high-resolution crystal structures presented here, we now have a detailed understanding of their mode of action, which will in turn, inform the discovery and development of alternative scaffolds, and may eventually lead to the conversion of this highly useful ‘tool compound’ into a potential bona fide drug candidate for the treatment of disease.

## ACCESSION NUMBERS

5J3P, 5J3S, 5J3Z and 5J42 for apo-hTDP2^CAT^, hTDP2^CAT^+163, m2hTDP2^CAT^+163 and m2hTDP2^CAT^+148 respectively.

## Abbreviations

DDR: DNA damage response
DSB: double-strand break
EAPII: ETS1-associated protein II
HTS: high-throughput screen
PARP1: poly(ADP-ribose) polymerase 1
SAR: structure–activity relationship
SENP1: sentrin-specific protease 1
SUMO: small ubiquitin-like modifier
TDP2: tyrosyl-DNA phosphodiesterase 2
*T*_m_: temperature midpoint
TOP2: topoisomerase II
TTRAP: TRAF and TNF receptor-associated gene
